# Antioxidant Effect of *Ocimum basilicum* Essential Oil and Its Effect on Cooking Qualities of Supplemented Chicken Nuggets

**DOI:** 10.3390/antiox11101882

**Published:** 2022-09-23

**Authors:** Hafiz Rehan Nadeem, Saeed Akhtar, Tariq Ismail, Muhammad Qamar, Piero Sestili, Wisha Saeed, Muhammad Azeem, Tuba Esatbeyoglu

**Affiliations:** 1 Institute of Food Science and Nutrition, Bahauddin Zakariya University, Multan 60800, Pakistan; 2 Department of Biomolecular Sciences, Università degli Studi di Urbino Carlo Bo, 61029 Urbino, Italy; 3 Department of Chemistry, COMSATS University Islamabad, Abbottabad Campus, Abbotabad 22060, Pakistan; 4 Department of Food Development and Food Quality, Institute of Food Science and Human Nutrition, Gottfried Wilhelm Leibniz University Hannover, Am Kleinen Felde 30, 30167 Hannover, Germany

**Keywords:** antioxidant, basil, essential oil, functional food, meat, microbial spoilage, preservative, sensory, shelf life, toxicity

## Abstract

A commonly observed chicken meat issue is its lipid oxidation that leads to deterioration of its organoleptic and nutritional properties and its further-processed products. Basil *(Ocimum basilicum* L.) is one of the traditional culinary herbs exhibiting food preservation properties. The current study investigated the essential oil composition, antioxidant activity and in vitro cytotoxic capacity of the essential oil of basil indigenous to Pakistan. GC–MS analysis of the essential oil revealed the presence of 59 compounds that constituted 98.6% of the essential oil. *O. basilicum* essential oil (OB-EO) exhibited excellent antioxidant activity, i.e., IC_50_ 5.92 ± 0.15 µg/mL as assayed by the DPPH assay, 23.4 ± 0.02 µmoL Fe/g by FRAP, and 14.6 ± 0.59% inhibition by H_2_O_2._ The brine shrimp lethality assay identified an average mortality of ~18% with OB-EO at 10–1000 µg/mL, while that of the same concentration range of the standard drug (etoposide) was 72%. OB-EO was found to be non-toxic to HeLa and PC-3 cell lines. TBARS contents were significantly decreased with increase of OB-EO in chicken nuggets. The lowest TBARS contents were recorded in nuggets supplemented with 0.3% OB-EO, whereas the highest overall acceptability score was marked to the treatments carrying 0.2% OB-EO. The results suggest OB-EO as a promising carrier of bioactive compounds with a broad range of food preservation properties, and which has a sensory acceptability threshold level for chicken nuggets falling between 0.2-0.3% supplementation. Future research must investigate the antibacterial impact of OB-EO on meat products preserved with natural rather than synthetic preservatives.

## 1. Introduction

Meat and meat-based products are generally considered nutrient-rich food products and ranked in the food guide pyramid as well [1]. In contrast, chicken meat, being low in fat content, is globally most acceptable, represents a widely consumed meat group and is expected to be ranked the highest over the next few years [2]. Although poultry meat is a low calorie and low-fat content meat, its high degree of unsaturation of muscle lipids makes chicken products highly susceptible to oxidation. However, chicken meat’s popularity is attributed to its economic and physical accessibility without any religious or cultural limitations toward its consumption as reported for beef or pork meat. Healthier nutritional value, low price, easy availability, and incorporation in processed foods make poultry meat preferable in the modern era [3].

As was anticipated, a commonly observed chicken meat issue is its lipid oxidation that leads to deterioration of organoleptic and nutritional properties of meat and meat products. Intrinsic factors impacting on oxidative balance, such as the level of antioxidant enzymes or the iron content, and extrinsic factors such as environmental stress, oxidized feed, slaughtering procedure, storage conditions, and diseases have been reported to play a significant role in producing oxidative stress in chicken meat [4]. Currently, food regulatory authorities are highly concerned with the public health aspects of food safety, and also whether consumers have enough knowledge of food safety. Many reports on the short- and long-term toxicity of synthetic preservatives are also a major constraint limiting their use, especially in meat-based perishable food products. Cooked meat products, on account of their ideal nutrient composition, have susceptibility toward oxidative and microbiological contamination. Synthetic antioxidants and antimicrobial compounds have wide industrial applications to maintain the quality and safety of raw and cooked meat products. However, despite having lower effective application levels, synthetic antioxidants and antimicrobials are debated as being hazardous to consumer health on consistent consumption [5,6,7].

To meet the continuously increasing demand of customers for meat-based, ready-to-cook products, the global meat industry is growing in volume to cover this demand. Increased customer knowledge regarding safe and nutritious food has attracted the attention of the research community to explore natural and risk-free food preservatives as replacements to synthetic ones. Recent research databases suggest that extracts of some plant parts and their essential oils are effective against meat pathogens as synthetic preservatives [8]. In this regard, extensive research has been carried out, especially on aromatic plants carrying pools of bioactive compounds bearing antioxidant properties. The evidence suggests that these natural plant-based preservatives are least- to non-toxic, and improve the storage stability and physiological functionality of meat products. Interestingly, the research evidence suggests that the bioactive compounds of aromatic plants have proven health-promoting properties if used as a value-added ingredient in food product development [9,10]. Considering all facts critical to human health, natural preservatives are a cheap and healthier alternative to synthetic additives, and are encouraged to be used in the meat industry. Researchers have validated natural preservatives of plant origin to offer stronger antioxidant and antimicrobial properties than conventional additives, such as sodium nitrate [6,11]. Basil is one of the culinary plants traditionally used as a preservative due to its unique bioactive composition. The essential oils of basil predominately extracted from the leaves have diverse uses/applications, such as an additive, preservative, and a therapeutic ingredient in foods of plant and animal origin [12].

*Ocimum basilicum* L., commonly known as basil (the Greek word Basileus meaning royal/king), is referred to as the king of herbs [13]. Basil has been well-known from ancient times and is mostly used as a therapeutic ingredient in Unani and Ayurvedic medical systems [14]. Basil is, interestingly, indigenous to Pakistan, and has applications which include, but are not limited to, traditional medicine, culinary use, essential oil production, and as a value-added ingredient for the development of drinks in summer [15]. Traditionally, its oil is extracted from the leaves and flowers, and is extensively used in food processing systems as a flavoring agent and in pharmaceutical industries for medicinal purposes [16]. The composition of basil essential oil significantly varies with the seasonal variation, growing region/location, and development stages. However, OB-EO mainly contains oxygenated and aromatic oxygenated monoterpenes, eucalyptol, linalool, eugenol, methyl-chavicol, geraniol, methyl cinnamate, τ-cadinol, camphor, and carvacrol [17,18]. Recent research evidence on essential oils with unique interesting bioactive and fatty acid composition has drawn the attention of researchers to investigate its effectiveness as a food additive and its versatility in industrial applications. OB-EO has been reported to anticipate preservative effects in beef (antioxidant) and chicken sausages (antibacterial), respectively [19,20].

A gap exists indicating the need to transform conventional knowledge on the application of OB-EO as an aesthetic flavoring to an exploration of its food preservation properties. There are few reports available on basil supplementation in foodstuffs to anticipate the stability and functional properties of the consumer good. Tongnuanchan et al. (2014) [21] described the application of OB-EO in fish gelatin films to maintain the fish quality. Still, there are no studies recognizing the use of OB-EO as a natural preservative in ready-to-cook meat products, such as nuggets, and as a functional ingredient in value-added foodstuffs. Against this backdrop, the essential oils of sweet basil, after being explored for their bioactive compounds, safety and antioxidant properties, were evaluated for suitability as a natural preservative and flavoring ingredient in chicken nuggets.

## 2. Materials and Methods

### 2.1. Collection and Preparation of Raw Material

Basil leaves were obtained from mature basil plants cultivated in controlled conditions in a local horticulture nursery in Multan, Punjab, Pakistan. The leaves were washed with potable water, drained and dried in shade to a moisture content of less than 10% (mean temperature 31 °C, mean relative humidity 33%). The dried leaves were ground to a powder in a kitchen grinder and packaged in polyethylene zip bags, and stored at −20 °C for later use.

### 2.2. Hydro-Distillation Extraction of Essential Oil

The essential oil was extracted by the hydro-distillation technique as adopted by Abbas et al. (2022) [22]. Fifty grams of the dried ground leaves were taken in a 1000 mL flask connected to the Clevenger apparatus. In the same flask, 750 mL of distilled water was added. The sample was allowed to undergo hydro-distillation for 4 h. The distilled essential oil was then collected and dried over anhydrous sodium sulphate, filtered, and stored in sealed vials at −18 °C for further analyses.

### 2.3. GC–MS Analysis of Essential Oil

A Hewlett Packard (HP) GC–MS (gas-chromatography–mass-spectrometry) system (Agilent Technologies Inc., Santa Clara, CA, USA) was used to analyze the essential oils. The GC–MS parameters were adopted from Azeem et al. (2019) [23] with modifications. The 6890N GC was equipped with a DB-5 capillary column (30 m long, 0.25 mm internal diameter, and 0.25 µm stationary phase film thicknesses). The GC injector was isothermally set at 235 °C. The GC oven temperature was programmed as follows: the initial temperature was held isothermally at 40 °C for 2 min, then raised to 240 °C at the rate of 5 °C/min. The temperature was then ramped to 280 °C at the rate of 20 °C per min and set at the highest temperature for 0 min. Helium gas was used as a mobile phase with a constant flow rate of 1 mL/min. A diluted solution of essential oil was injected with a volume of 1 µL in a splitless mode for 30 s. The parameters for the mass spectrometer were as follows: the electron ionization was carried out at 70 eV, the ion source temperature of the MS was isothermally set at 180 °C and the filament off time for 5 min, and the mass spectra scan range was *m/z* 30–400. Total ion chromatogram peak areas were used to calculate each compound’s percentage composition in the essential oil. The identification of the separated compounds was initially carried out by comparing their mass spectra with the NIST-2008 MS library. Additionally, the retention indexes of separated compounds were determined relative to the retention times of a series of *n*-alkanes (C_8_–C_24_) analyzed with the same GC–MS parameters used for the essential oil. The calculated retention indexes of separated compounds were compared with published data to determine the elution order and identify compounds [24]. Finally, wherever possible, the identification of a compound was verified by injecting an authentic, pure standard compound purchased from Sigma-Aldrich (Stockholm, Sweden) at the same parameters used for essential oil analysis.

### 2.4. Evaluation of Antioxidant Potential

Antioxidant potential was evaluated using three different assays, i.e., DPPH, H_2_O_2_, and FRAP assay as adopted by Qamar et al. [25,26]. In DPPH and H_2_O_2_ assays, distilled water was used as a blank, quercetin (125 μg/mL) as standard, and the findings were reported as IC_50_ μg/mL and percent inhibition, respectively. In the FRAP assay, ferrous sulphate was used for calibration. Results were expressed as Fe µmoL/g.

### 2.5. Cytotoxicity Assessment

#### 2.5.1. Brine Shrimp Lethality Test

The brine shrimp eggs were hatched in artificial seawater and used after 48 h. The lethality test assay was carried out as described by Meyer et al. (1982) [27]. The samples at different concentrations (10, 100, and 1000 µg/mL) in triplicate were transferred in glass vials and then evaporated. Next, artificial seawater was added to each vial to achieve the correct concentration. Thirty shrimps were added to each vial, and the number of dead shrimps per dose was recorded after 24 h. The survival rate of the shrimps in all vials including positive control, i.e., etoposide, was counted.

#### 2.5.2. 3-(4,5-Dimethylthiazol-2-yl)-2,5-diphenyltetrazolium Bromide (MTT) Assay

The cytotoxic response of OB-EO against HeLa and PC-3 cell lines was performed as suggested by Roy et al., (2002) [28]. HeLa (human cervical carcinoma; ATCC CCl-2) and PC-3 (human prostate cancer; ATCC CRL-1435) cell lines were obtained from the Husain Ebrahim Jamal Research Institute of Chemistry (HEJ), Karachi/Pakistan. Test samples (OB-EO) were prepared as 0.5–200 μg/mL in 1% dimethylsulphoxide (DMSO) and diluted to a final concentration between 0.5–200 μg/mL in microtiter plate wells. The incubation (5% CO_2_) of the microtiter plate was performed at 37 °C for 48 h, and 50 μL of the MTT solution (5 mg/mL) was added to each well. The second incubation was performed in darkness at 37 °C for 4 h. Absorbance was measured at 570 nm in a microplate reader (Tecan Infinite M200, Männedorf, Switzerland). The untreated cells (0.1% DMSO) were used as a control against which to measure the effect of experimental extracts on the cell viability. The percent inhibition exhibited on the cell cultures by the test samples was computed using the following equation:

Survival (%) = (At − Ab)/(Ac − Ab) × 100

where At, Ab, and Ac indicate the test samples, blank (complete media without cells), and control (untreated cells) absorbance, respectively.

Cell inhibition (%) = 100 − cell survival (%)

### 2.6. Nugget Preparation

Chicken nuggets were prepared according to the commercial recipe of the Dawn Foods processing company (Lahore, Pakistan). For this purpose, four uniform formulations of chicken batter were prepared by mixing skinless, boneless breast (54% *w/w*), premium skin (20% *w/w*), water (22% *w/w*), sodium tripolyphosphate (STTP) (0.6% *w/w*), salt (0.7% *w/w*), garlic powder (0.2% *w/w*) and vitacel (2% *w/w*). In addition, premium skin was replaced with 0.1% EO, termed as treatment 1 (T_1_), 0.2% EO (T_2_), and 0.3% EO (T_3_) in separate formulations to determine the effect of oil supplementation in nuggets. For the control (T_0_), a mixture without additional essential oil was used. Each blend was mixed gently in a bowl for 5 min to attain the homogeneous mixture. The brick-shaped nuggets were obtained by slicing (4 × 2 × 1 cm). Raw nuggets were stored at −18 °C for 45 min and then coated with a mixture of milk, egg, and bread crumbs. Finally, nuggets were fried at 80 °C for 3 min for uniform cooking. Fried nuggets were packed in polythene bags and stored at 4 °C for storage studies at 0, 7, and 14 days.

#### 2.6.1. Sensory Evaluation

Products were evaluated for sensory characteristics, i.e., color, taste, texture, and overall acceptability in the sensory evaluation laboratory by a panel of 10 judges (8 men and 2 women; aged between 35–60 and 32–45, respectively) on a 9-point hedonic scale [29].

#### 2.6.2. Proximate Composition

Ash, moisture, fat content and protein of cooked nuggets were assessed according to the approved methods of AOAC [30].

#### 2.6.3. Cooking Properties

The cooking yield was determined as reported by Naveena et al. (2006) [31] as follows:

Cooking Yield (%) = (weight of cooked nuggets/weight of raw nugget) × 100

Fat retention was calculated according to Murphy et al. (1975) [32] using the following equation:

Fat retention (%) = (cooked weight × % fat in cooked nuggets)/(raw weight × % fat in raw nuggets) × 100

Moisture retention was determined according to El-Magoli et al. (1996) [33] as follows:

Moisture retention (%) = (moisture in cooked nuggets (%)/moisture in raw nuggets (%)) × cooking yield (%)

#### 2.6.4. Evaluation of pH

The pH of chicken meat nuggets was measured according to Troutt et al. (1992) [34] using the combined glass electrode of an Elico pH meter (Model LI 127, Mumbai, India).

#### 2.6.5. TBARS Analysis

Lipid oxidation was measured using the determination of thiobarbituric acid reactive substances (TBARS) by a modified version of the procedure reported by Kerth and Rowe (2016) [35] using a microplate reader (Synergy HT Multi-Mode Microplate Reader, Biotek, Winooski, VT, USA). TBARS values were expressed as mg malondialdehyde equivalents per kg of chicken nuggets.

#### 2.6.6. Color Consistency

Color measurement was carried out using a Hunter Colorimeter model 45/0-L mini scan XE PLUS (Hunter Associates Labs, Reston, VA, USA) based on three variables, namely, L, a, and b. The instrument was calibrated against a standard blank as well as white reference tiles. The samples were placed in a transparent petri dish and positioned directly on the light path to measure the color parameter values of L, a, and b. Four color readings were taken from each chicken nugget sample, and the average value was used for analysis [36].

#### 2.6.7. Texture Analysis

Texture profile analysis (TPA) was performed at 25 °C with a Texture Analyzer STM20 (Santam, Tehran, Iran). Samples (1 cm thickness and 1.5 cm diameter) were cut from nuggets and exposed to a compression two-cycle test. Compression was completed to 50% of the original height of nuggets with a diameter (2.5 cm) cylindrical probe at a 5–50 kg load of compression and a 1.6 mm/s cross-head speed. Parameters of texture profile analyzed were hardness, springiness, gumminess, and chewiness [37].

### 2.7. Statistical Analysis

All data were expressed as mean ± standard error (S.D). The statistics applied were Student’s *t*-test and analysis of variance (ANOVA), and *p* < 0.05 was considered significantly different using GraphPad Prism (Graph Pad Software, V8, San Diego, CA, USA, http://www.graphpad.com, accessed on 12 March 2021).

## 3. Results

### 3.1. Composition of the Essential Oil

GC–MS analysis of the essential oil revealed the presence of fifty-nine compounds, wherein the major compounds were estragole (52.3%), linalool (15.7%), *trans*-α-bergamotene (7.29%) and 1,8-cineol (5.56%). These major compounds represented 80.9% of the essential oil overall composition (Table 1).

### 3.2. Antioxidant Potential

In this study, the antioxidant activity of basil essential oil was evaluated using three different assays: DPPH, FRAP and H_2_O_2_. OB-EO exhibited remarkable antioxidant activity in DPPH (IC_50_ 5.92 µg/mL), FRAP (23.4 µmoL Fe/g), and H_2_O_2_ (14.6% inhibition) assays (Table 2). It can be stated that high antioxidant activity of the OB-EO may potentially relate to the bioactive compounds reported in Table 1.

### 3.3. Cytotoxicity Assessment

#### 3.3.1. Brine Shrimp Lethality Test

Basil essential oil at the tested concentrations, i.e., 10, 100, and 1000 µg/mL did not show any toxicity, with the LC_50_ value being higher than 1000 µg/mL (Table 3). The average mortality of the brine shrimps exposed to OB-EO at 10–1000 µg/mL was 17.6%, while the average mortality rate of shrimps on similar doses of etoposide (standard drug/control) was 72.3%.

#### 3.3.2. MTT Assay

OB-EO at 30 µg/mL recoded to induce non-substantial cytotoxicity. The cytotoxic response of OB-EO at 30 µg/mL against HeLa and PC-3 cell lines was 29.7% and 8.9% inhibition, respectively (Table 4). Doxorubicin was used as the standard drug and showed 92.2% and 89.9% inhibition at the same concentration against HeLa and PC-3 cell lines, respectively. These results revealed the inactive behavior of OB-EO, which was found to be non-toxic to both tested cell lines.

### 3.4. Sensory Evaluation

The results for the organoleptic evaluation of OB-EO-supplemented nuggets are shown in Table 5. The results indicated that there was a variable impact of OB essential oil supplementation on different sensory attributes of the nuggets. However, all treatments, except for those with 0.3% essential oil, were reported as acceptable for different sensory attributes by the sensory panelists. Contrary to the other sensory attributes, the highest score for color was given to the nuggets prepared with 0.3% OB-EO, followed by the control treatment (T_0_). Similarly, the highest score for flavor was given to the nuggets carrying 0.2% essential oil, followed by the control and the treatment with 0.1% essential oil, which differ non-significantly from each other. The lowest flavor score was assigned to the nuggets prepared by supplementation of 0.3% OB-EO (T_3_). Among other sensory parameters, the highest mouthfeel and a significantly different score was recorded for the nuggets carrying 0.2% OB-EO, followed by T_1_, whereas the lowest score was given to the nuggets prepared with 0.3% OB-EO (T_3_). Taste, texture and overall acceptability scores were also recorded highest for the T_2_, followed by the control and T_1_, while significantly lower scores were given to nuggets carrying 0.3% OB-EO.

### 3.5. Proximate Composition and Cooking Properties

The results for proximate composition of OB-EO-supplemented nuggets are given in Table 6. Mean moisture, crude fat, crude protein and ash contents for different treatments and the control ranged between 43.38 to 45.67%, 32.09 to 32.20%, 15.82 to 15.80% and 1.77 to 1.78%, respectively. The results of the cooking properties of the nuggets are also available in Table 6. The cooking yield from the nuggets supplemented with 0.1–0.3% OB-EO and the control ranged between 81.7 to 85.3%, whereas the moisture and fat retention capacity of the control and treatments ranged between 60.2 to 62.3% and 83.8 to 88.6%, respectively (Table 6).

### 3.6. Evaluation of pH

The pH of any food product plays a major role in consumer acceptability, as well as the shelf life of the product. In the current study, the pH of the nuggets was found to decrease with the increase of OB-EO. However, periodic testing results showed that the pH of the nuggets increased during 14 days of refrigerated storage, from 5.28 to 5.78 on the 14th day (Table 7).

### 3.7. TBARS Analysis

Formation of TBARS is a common storage issue in oil-based processed foods that causes oxidative rancidity, and is a prime reason for the reduced storage stability of such products. The results regarding supplementation of OB-EO in the nuggets demonstrated that TBARS contents were significantly decreased with increasing OB-EO supplementation levels in chicken nuggets. TBARS content increased over 14 days of storage (Table 7). The highest TBARS value was recorded for the control nuggets samples, while the lowest values of TBARS were observed for the treatment carrying 0.3% OB-EO. Similarly, the highest TBARS value, i.e., 1.11 mg MDA/kg, was observed in the chicken nuggets stored for 14 days, while the lowest TBARS value (0.32 mg MDA/kg) was recorded at the beginning of storage.

### 3.8. Color Consistency

Color is one of the most important visual quality attributes of oil-based processed (fried) foods. The color of the fried product changes according to the type of oil/fat used in frying and the degree of frying. Our results suggest a reduction in the degree of the lightness (L) of the nuggets prepared with OB-EO, while it also decreased during the 14 days of storage. In the case of redness (a), the values decreased with the addition of OB-EO, whereas an increase in the degree of redness was observed during the 2 weeks of storage. The yellowness (b) value of the supplemented nuggets also increased with the addition of OB-EO and the product storage (Table 7).

### 3.9. Texture Analysis

The effect of the storage period and treatment on the hardness and gumminess of OB-EO-supplemented nuggets was found to be non-significant. However, means comparison of the textural parameters of the OB-EO-supplemented nuggets for the treatments and storage days demonstrated that springiness, cohesiveness and chewiness were significantly influenced (Table 8). A non-significant change in hardness and the gumminess of the nuggets was noticed with OB-EO supplementation and over the storage duration. The results also indicate a non-significant combined effect of treatment and storage days on all textural parameters of the nuggets.

## 4. Discussion

In the present study, OB-EO exhibited excellent antioxidant activity in DPPH, FRAP, and H_2_O_2_ assays (Table 2). It can be stated that high antioxidant activity could be attained by adding the bioactive compounds quantified in this study. A plethora of literature showed a positive relationship between specific bioactive compounds and antioxidant activity [38,39,40,41,42,43,44]. In an earlier study, the antioxidant activity of OB-EO was reported as IC_50_ 6.68 mg/mL and 62.2 mg ascorbic acid equivalent (AAE)/g in DPPH and FRAP assay, respectively, and was ascribed to the presence of α-pinene, γ-terpinene, 1,8-cineole, limonene, linalool, and linalyl acetate [45], supporting the findings of the present study. In another study conducted by Hanif et al. (2017) [46], the antioxidant potential of basil oil was reported, with a % inhibition of 60.6, 19.6, and 88.2 in DPPH scavenging assay, H_2_O_2_ scavenging assay, and in linoleic acid system, respectively. Furthermore, Hussain et al. (2008) [22] recorded the excellent antioxidant activity of basil essential oil in DPPH assay (IC_50_ 4.8–6.7 μg/mL) and in linoleic acid system (% inhibition 84.3–91.3) in accordance with the standard butylated hydroxytoluene (BHT), wherein linalool contents were calculated as 56.7%. OB-EO was also able to reduce the stable violet/stable DPPH radical to yellow colored DPPH-H [47]. The findings of the current investigation showed no or at least negligible toxicity of OB-EO, in contrast with the findings of Sharopov et al. (2016) [48], who noticed active cytotoxic behavior of OB-EO (LC_50_ 3.19 ± 0.84 µg/mL) while LC_50_ to positive control was 6.33 ± 3.35 µg/mL. However, Fandohan et al. (2008) [49] observed acute toxicity of OB-EO in an animal model, and a LD_50 of_ 3250 mg/kg body weight in rats was recorded. The variability in the toxicological facts of OB depends on various factors, including the cultivar, the height and age of the plant, the heterogenicity of the biochemical composition of plant material, type of extract, the part of the plant tested, and the chemotype (Sestili et al., 2018) [50]. In the present study, OB-EO exhibited low or no cytotoxicity against HeLa and PC-3 cell lines, respectively. The results of the present study are in keeping with the findings of Rezzoug et al. (2019) [51], who reported OB-EO LD_50_ values of 1052  ±  38 and 1028  ±  78 µg/mL against HeLa and PC-3 cell lines, respectively. Hence, the findings of our study show support for the supplementation of basil in foodstuffs as a safe ingredient for consumption, as OB-EO showed considerably low cytotoxic activity.

Table 5 shows the organoleptic findings of OB-EO supplemented nuggets. OB-EO supplementation had varying effects on nugget sensory parameters. The influence of essential oils added for antimicrobial and/or preservative purposes on foods’ organoleptic physiognomy is not easily predictable, but obviously needs to be addressed case by case. For example, a previous report by Ahmed et al. indicated that the addition of 0.3% of orange peel essential oil did not affect the sensory evaluation of the cupcake (Ahmed et al., 2009) [52], while in another study by Ibrahium et al., the sensory evaluation revealed that the sample containing 800 ppm of clove essential oil showed the lowest acceptability score in cakes, in addition to preservation effects (Ibrahium et al., 2013) [53]. Our results indicate that OB-EO supplementation at 0.1–0.2% did not alter the overall acceptability of the chicken nuggets, while increasing the level of supplementation to ࣙ0.3% resulted in undesirable changes in taste, flavor and mouthfeel responses of the chicken nuggets.

The proximate composition and cooking properties of nuggets supplemented with OB-EO were almost similar to the control nuggets (Table 6), thus suggesting that supplementation with 0.1–0.3% OB-EO does not impart any change in nutritional composition and cooking properties. A non-significant impact of black cumin essential oil on the nutritional profile of cereal-based bakery products was reported in a previous study by Sultan et al. (2012) [54]; an opposite trend in moisture, protein, and fat contents was observed, but the variation was negligible when compared to the present study. Yogesh et al. (2013) [36] conducted a research study on chicken nugget characteristics with variable added fat and salt contents. The authors reported that cooking yield improved from 88.0 ± 0.2 to 89.3 ± 0.36%, and attributed the improvement to added fat. The same study also reported that moisture retention values ranged from 70.9 ± 0.57% to 79.4 ± 0.73% in chicken nuggets with variable fat and salt contents.

In this study, it was found that increasing the level of OB-EO supplementation decreased the pH of the nuggets. It has been previously reported that essential oil supplementation at 0.1-10% in nutrient broths reduced the pH of the substrate from 7.29–5.2 [55], while, interestingly, weekly (0, 7 and 14 days) testing of the nuggets showed an increasing trend in their pH over time (Table 7). The increase in pH of the chicken nuggets over 14 days under refrigerated storage conditions is likely associated with lactic acid and protein degradation [56]. The results are in line with the findings of Bhat et al. (2015) [57] and Kaur et al. (2015) [58], who also reported a decline in the pH of the nuggets due to the presence of bioactive compounds. Similarly, compared to non-supplemented nuggets, the pH value of nuggets supplemented by OB-EO increased during the storage period. This increment in pH of the products during storage might be due to hydrolytic (enzymatic and acid) breakdown/conversion of larger molecules into acidic small units. These results are in accordance with those of Mokhtar et al. (2014) [59], who also reported an incremental increase of pH in nuggets during storage due to the conversion of proteins into amino acids. The results are also in keeping with the findings of Kumar and Tanwar (2011) [60], Sudheer et al. (2011) [61], Tanwar et al. (2016) [62], Zhang et al. (2016) [63], Sharma et al. (2017) [64] and Wanangkarn et al. (2018) [65], who reported that the pH of the meat products increased during the storage period. As for TBARS content in nuggets, we found a progressive increase, likely due to the oxidation of lipids, over the storage period [66,67]. The results are in line with the findings of Zargar et al. (2014) [68], Kaur et al. (2015) [58], Tanwar et al. (2016) [62] and Wanangkarn et al. (2018) [65], who also reported that the TBARS of the meat products increased due to lipid oxidation with the passage of time. However, it is worth noting that although TBARS also increased in OB-EO-supplemented nuggets, the increase was lower and, differently from the controls, did not overcome the acceptable limits of 1.00 mg MDA/kg at the 14th day of the storage period in T_1_, T_2_ and T_3_ nuggets. Hence, these results clearly indicate that OB-EO exerts remarkable antioxidant activity capable of reducing the lipid oxidation of the nuggets. The results are supported by a number of other authors, who also reported that *O. basilicum* leaves possess strong antioxidant activity [46,69,70]. The darkness of the product reveals the fact that added oil involves the complex interactions between the fatty acids of OB-EO with the fatty acids of other oil and polymers of fried product. Jaswir et al. (2000) [71] reported that non-polar components of fried food products leached down in the oil, forming polymerized complexes that resulted in the darkening of medium oil and its reflection on fried products. Recent evidence also proposed that during frying, various fried food components react with frying oil components, while some other volatile components generate due to thermal oxidation, and that collectively all these components and their interactions resulted in the darkening of fried oil [72]. The change in the color of nuggets might also be due to the natural yellow-green color of basil leaves. The results are justified with the findings of Park et al. (2006) [73], Sáyago-Ayerdi et al. (2009) [74], Hwang et al. (2013) [75], Yogesh et al. (2013) [36] and Sharima-Abdullah et al. (2018) [76], who also observed the variation in lightness, yellowness and redness of different meat products with the addition of natural extracts and powder. Supplementing OB-EO did not lead to significant changes in the degree of the nuggets’ hardness and gumminess. Aging could be the factor behind slight changes in the means of the treatments for springiness, cohesiveness and chewiness during the 14 days of storage. Previously, essential oils have been documented to attribute an improvement in various textural properties of ready-to-cook meat-based products by reducing hardness and other associated parameters [4].

Another study by Madane et al. (2019) [77] reported an extract of *Moringa oleifera* flowers to not significantly influence the texture properties of chicken meat nuggets. The current results are also in accordance with the findings of Estévez et al. (2005) [6], Lara et al. (2011) [78], Andrés et al. (2017) [79], Cunha et al. (2018) [80], and Wanangkarn et al. (2018) [65], who also noticed the change in textural properties of meat products as an aging phenomena. However, there were non-significant changes in many of the textural attributes of chicken nuggets under EO-OB supplementation, though the essential oils of the leaves of *O. basilicum* were found to be promising in inhibiting lipid oxidation, and in imparting pleasing sensory attributes to the finished edible good. In summary, our work on EO-OB as a functional ingredient in improving the sensorial attributes and keeping quality of chicken nuggets validate earlier research suggesting EO-OB supplementation at lower doses as a natural antioxidant, flavor enhancer and color improver in meat-based preparations [19,81].

## 5. Conclusions

The major compounds of *O. basilicum* are 1,8-cineol (5.56%), linalool (15.7%), estragole (52.3%) and *trans*-α-bergamotene (7.29%). High antioxidant activity observed in this study was linked with the presence of bioactive compounds in the essential oil. OB-EO induced no to negligible toxicity in a brine shrimp lethality assay, and cytotoxicity in MTT assay. The addition of 0.3% OB-EO (T_3_) improved the color of nuggets as compared to the control (T_0_), whereas the highest scores for flavor, mouthfeel, taste, texture, and overall acceptability were given to nuggets supplemented with 0.2% OB EO (T_2_), and the lowest scores were given to T_3_. The results regarding supplementation of OB-EO in the nuggets demonstrated that TBARS contents were significantly decreased with the increase of OB-EO dose level in the supplemented nuggets. The highest TBARS value was detected in T_0_, while the lowest was detected in T_3_. Similarly, the highest TBARS value was found after the 14th day of storage, while the lowest TBRAS value was shown at the beginning of storage. Collectively, the aforementioned results support the supplementation of *O. basilicum* in nuggets as a safe ingredient for consumption. Future studies must be conducted with varying concentrations of OB-EO in order to achieve sensory characteristics comparable to commercial chicken nuggets. Future studies must be conducted investigating the antibacterial effect of OB-EO on meat products, aiming at natural preservation instead of synthetic preservatives.

## Figures and Tables

**Table 1:** Chemical composition of *O. basilicum* essential oil determined by GC–MS analysis.

Identified Compound	Determined RI	Reported RI	Composition %
β-Myrcene	988	988	0.46
**1,8-Cineole**	1031	1031	5.56
*trans*-β-Ocimene	1047	1047	0.45
**Linalool**	1104	1104	15.7
Camphor	1146	1144	1.40
α-Terpineol	1191	1192	0.62
**Estragole**	1197	1195	52.3
Bornyl acetate	1288	1288	0.96
Methyleugenol	1406	1401	0.67
β-*trans*-Caryophyllene	1426	1423	0.53
**trans-α-Bergamotene**	1442	1438	7.29
α-Farnesene	1490	1506	0.47
δ-Cadinene	1528	1525	0.47
Cubenol	1622	1628	0.48
τ-Cadinol	1649	1642	3.86

**Table 2:** Antioxidant potential of *O. basilicum* essential oil.

Sample	FRAP (µmoL Fe/g)	DPPH (IC_50_ µg/mL)	H_2_O_2_ (Inhibition %)
Basil essential oil	23.4 ± 0.02	5.92 ± 0.15	14.6 ± 0.59
Quercetin	34.6 ± 0.10	4.96 ± 0.21	72.3 ± 0.10

Values are means ± S. D. of three measurements. IC_50_, The half-maximal inhibitory concentration.

**Table 3:** Brine shrimp lethality assay for *O. basilicum* essential oil (OB-EO).

Sample	No. of Shrimps	No. of Survivors at Various Concentration of Extracts			Average Mortality %
		10 µg/mL	100 µg/mL	1000 µg/mL	
Essential Oil	30	27	24	23	17.6
Etoposide	30	14	09	02	72.3

OB-EO: *O. basilicum* essential oil.

**Table 4:** MTT assay for OB essential oil.

Sample	Concentration (µg/mL)	HeLa		PC3	
		Inhibition (%)	IC50	Inhibition (%)	IC50
Essential Oil	30	29.7	-	8.9	-
Std. Doxorubicin	30	96.2	0.4 ± 0.07	89.9	1.9 ± 0.08

OB: *O. basilicum*. IC50: The half-maximal inhibitory concentration.

**Table 5:** Sensory evaluation of nuggets supplemented with *O. basilicum* essential oil on a 9-point hedonic scale.

Treatments	Color	Taste	Texture	Flavor	Mouthfeel	Overall Acceptability
T_0_	6.67 ± 0.58AB	7.83 ± 1.04AB	6.5 ± 1.32AB	7.5 ± 0.5B	7.61 ± 0.53AB	7.19 ± 0.56AB
T_1_	6.05 ± 0.09C	7.87 ± 0.32AB	6.5 ± 0.87AB	7.53 ± 0.9B	7.5 ± 0.5B	7.22 ± 0.26AB
T_2_	6.57 ± 0.51B	7.92 ± 0.38A	6.83 ± 0.76A	7.75 ± 0.66A	7.67 ± 0.58A	7.35 ± 0.4A
T_3_	6.77 ± 0.67A	6.33 ± 1.15B	6.17 ± 1.26B	6.67 ± 0.58C	6.73 ± 0.64C	6.53 ± 0.57B

T_0_: Control, T_1_: 0.1% EO, T_2_: 0.2% EO, T_3_: 0.3% EO. Different letters between columns showed a significant difference between treatments.

**Table 6:** Proximate composition and cooking properties of *O. basilicum* essential oil-supplemented nuggets.

Treatment	Moisture (%)	Fat (%)	Protein (%)	Ash (%)	Cooking Yield (%)	Moisture Retention (%)	Fat Retention (%)
T_0_	43.4 ± 0.03	32.1 ± 0.01	15.9 ± 0.00	1.8 ± 0.00	85.3 ± 0.13	60.2 ± 0.09	88.6 ± 0.34
T_1_	44.0 ± 0.02	32.1 ± 0.6	15.8 ± 0.01	1.8 ± 0.01	84.7 ± 0.17	60.9 ± 0.08	86.2 ± 0.18
T_2_	44.1 ± 0.03	32.2 ± 0.5	15.8 ± 0.02	1.8 ± 0.02	83.4 ± 0.09	61.1 ± 0.10	84.3 ± 0.22
T_3_	45.7 ± 0.04	32.2 ± 0.03	15.8 ± 0.01	1.8 ± 0.01	81.7 ± 0.11	62.3 ± 0.07	83.8 ± 0.09

T_0_: Control, T_1_: 0.1% EO, T_2_: 0.2% EO, T_3_: 0.3% EO.

**Table 7:** Effect of *O. basilicum* essential oil supplementation on pH, TBARS, L, a and b value of chicken nuggets after refrigerated (4 °C) storage.

Treatments	0 Days	7th Day	14th Day	Mean
**pH**				
**T_0_**	5.45 ± 0.03e	5.87 ± 0.05b	6.21 ± 0.02a	5.84 ± 0.11A
**T_1_**	5.33 ± 0.05Fg	5.45 ± 0.05e	5.76 ± 0.02c	5.51 ± 0.07B
**T_2_**	5.23 ± 0.01g	5.34 ± 0.06f	5.62 ± 0.02d	5.40 ± 0.06C
**T_3_**	5.12 ± 0.05h	5.26 ± 0.02Fg	5.52 ± 0.01De	5.30 ± 0.06D
**Mean**	5.28 ± 0.04C	5.48 ± 0.07B	5.78 ± 0.08A	
**TBARS (mg malondialdehyde equivalents/kg)**				
**T_0_**	0.32 ± 0.01i	0.64 ± 0.02e	1.11 ± 0.01a	0.69 ± 0.12A
**T_1_**	0.28 ± 0.01ij	0.56 ± 0.02f	0.93 ± 0.03b	0.59 ± 0.09B
**T_2_**	0.25 ± 0.01jk	0.46 ± 0.01g	0.88 ± 0.02c	0.53 ± 0.09C
**T_3_**	0.21 ± 0.0k	0.37 ± 0.01h	0.78 ± 0.02d	0.45 ± 0.09D
**Mean**	0.27 ± 0.01C	0.51 ± 0.03B	0.92 ± 0.04A	
**Lightness (L)**				
**T_0_**	35.35 ± 0.62	34.32 ± 0.76	33.89 ± 0.98	34.52 ± 0.46A
**T_1_**	35 ± 0.81	34.00 ± 0.49	33.23 ± 1.15	34.08 ± 0.5A
**T_2_**	34.3 ± 0.69	32.32 ± 1.25	30.32 ± 0.63	32.31 ± 0.73B
**T_3_**	33.00 ± 0.9	31.21 ± 1.13	29.78 ± 0.68	31.33 ± 0.65B
**Mean**	34.41 ± 0.42A	32.96 ± 0.56B	31.81 ± 0.66B	
**Redness (a)**				
**T_0_**	3.90 ± 0.04e	4.20 ± 0.02c	4.50 ± 0.00a	4.2 ± 0.09A
**T_1_**	3.80 ± 0.06f	4.00 ± 0.02d	4.30 ± 0.01b	4.03 ± 0.07B
**T_2_**	3.67 ± 0.01g	3.88 ± 0.03e	4.23 ± 0.02bc	3.93 ± 0.08C
**T_3_**	3.65 ± 0.01g	3.76 ± 0.00f	4.00 ± 0.01d	3.8 ± 0.05D
**Mean**	3.76 ± 0.03C	3.96 ± 0.05B	4.26 ± 0.05A	
**Yellowness (b)**				
**T_0_**	20.12 ± 0.4	21.22 ± 0.76	22.3 ± 0.91	21.21 ± 0.48C
**T_1_**	21.00 ± 0.55	22.82 ± 0.42	23.73 ± 1.18	22.52 ± 0.56B
**T_2_**	21.32 ± 0.16	23.4 ± 0.58	24.30 ± 0.46	23.01 ± 0.49B
**T_3_**	23.43 ± 0.85	24.2 ± 0.66	25.43 ± 1.02	24.35 ± 0.52A
**Mean**	21.47 ± 0.44B	22.91 ± 0.42A	23.94 ± 0.52A	

T_0_: Control, T_1_: 0.1% EO, T_2_: 0.2% EO, T_3_: 0.3% EO. Different letters in columns showed a significant difference between treatments. L: Lightness. a: Redness. b: Yellowness.

**Table 8:** Effect of *O. basilicum* essential oil supplementation on textural properties of chicken meat nuggets at refrigerated (4 °C) storage.

Treatments	0 Days	7th Day	14th Day	Mean
**Hardness**				
**T_0_**	18.93 ± 0.57	19.08 ± 0.71	19.23 ± 0.85	19.08 ± 0.36
**T_1_**	18.90 ± 0.62	19.05 ± 0.76	19.20 ± 0.9	19.05 ± 0.39
**T_2_**	18.86 ± 0.66	19.01 ± 0.81	19.16 ± 0.95	19.01 ± 0.41
**T_3_**	18.83 ± 0.71	18.98 ± 0.86	19.13 ± 1.0	18.98 ± 0.44
**Mean**	18.88 ± 0.27	19.03 ± 0.34	19.18 ± 0.4	
**Springiness**				
**T_0_**	5.30 ± 0.08a	5.27 ± 0.08a	5.21 ± 0.06a	5.26 ± 0.04C
**T_1_**	5.37 ± 0.07a	5.34 ± 0.06a	5.28 ± 0.04a	5.33 ± 0.03BC
**T_2_**	5.44 ± 0.05a	5.41 ± 0.05a	5.35 ± 0.03a	5.4 ± 0.02AB
**T_3_**	5.51 ± 0.03a	5.48 ± 0.03a	5.42 ± 0.01a	5.47 ± 0.02A
**Mean**	5.40 ± 0.04	5.37 ± 0.03	5.32 ± 0.03	
**Cohesiveness**				
**T_0_**	0.44 ± 0.02	0.41 ± 0.01	0.37 ± 0.00	0.41 ± 0.01C
**T_1_**	0.46 ± 0.02	0.43 ± 0.01	0.39 ± 0.01	0.43 ± 0.01BC
**T_2_**	0.49 ± 0.03	0.45 ± 0.02	0.42 ± 0.01	0.45 ± 0.01AB
**T_3_**	0.51 ± 0.03	0.47 ± 0.02	0.44 ± 0.01	0.47 ± 0.02A
**Mean**	0.47 ± 0.01A	0.44 ± 0.01B	0.40 ± 0.01C	
**Gumminess**				
**T_0_**	8.35 ± 0.58	7.74 ± 0.45	7.12 ± 0.32	7.73 ± 0.29
**T_1_**	8.72 ± 0.72	8.12 ± 0.6	7.5 ± 0.46	8.11 ± 0.35
**T_2_**	9.18 ± 0.81	8.58 ± 0.69	7.97 ± 0.56	8.58 ± 0.39
**T_3_**	9.55 ± 0.96	8.96 ± 0.84	8.35 ± 0.71	8.95 ± 0.46
**Mean**	8.95 ± 0.36	8.35 ± 0.32	7.73 ± 0.27	
**Chewiness**				
**T_0_**	4.41 ± 0.24	4.07 ± 0.18	3.70 ± 0.12	4.06 ± 0.14C
**T_1_**	4.67 ± 0.33	4.32 ± 0.27	3.95 ± 0.21	4.32 ± 0.17BC
**T_2_**	4.98 ± 0.40	4.63 ± 0.34	4.26 ± 0.28	4.63 ± 0.2AB
**T_3_**	5.25 ± 0.50	4.90 ± 0.44	4.52 ± 0.38	4.89 ± 0.24A
**Mean**	4.83 ± 0.19A	4.48 ± 0.17AB	4.11 ± 0.15B	

T_0_: Control, T_1_: 0.1% EO, T_2_: 0.2% EO, T_3_: 0.3% EO. Different letters between columns showed a significant difference between treatments.

## Data Availability

The available data are presented in the manuscript.
